# Determining the predictive impact of donor parity on the outcomes of human leukocyte antigen matched hematopoietic stem cell transplants: a retrospective, single-center study

**DOI:** 10.3389/fonc.2024.1339605

**Published:** 2024-02-21

**Authors:** Mojtaba Azari, Maryam Barkhordar, Tanaz Bahri, Soroush Rad, Hosein Kamranzadeh Fumani, Seied Asadollah Mousavi, Sahar Tavakoli Shiraji, Morteza Azari, Parisa Shafaroudi, Mohammad Vaezi

**Affiliations:** ^1^ Cell Therapy and Hematopoietic Stem Cell Transplantation Research Center, Tehran University of Medical Sciences, Tehran, Iran; ^2^ Research Institute for Oncology, Hematology and Cell Therapy, Tehran University of Medical Sciences, Tehran, Iran; ^3^ Hematology, Oncology and Stem Cell Transplantation Research Center, Tehran University of Medical Sciences, Tehran, Iran; ^4^ Hematologic Malignancies Research Center, Tehran University of Medical Sciences, Tehran, Iran

**Keywords:** graft-versus-host disease (GVHD), hematopoietic stem cell transplantation (HSCT), donor parity, overall survival, relapse incidence

## Abstract

**Introduction:**

Donor choosing remains to play a pivotal role in allogeneic hematopoietic stem cell transplantation (allo-HSCT). Numerous criteria beyond HLA compatibility impact the selection of a suitable donor.

**Methods:**

We evaluated the effect of donor parity on transplant outcomes in a large homogeneously treated population that received an HLA-matched allo-HSCT between 2010 and 2021 at our center. All patients were transplanted from a peripheral blood stem cell source following a myeloablative Busulfan-based conditioning and an identical protocol for graftversus-host disease (GVHD) prophylaxis regimen.

**Results:**

A total of 1103 allo-HSCT recipients were included. 188 (17%) had transplants from parous female donors, whereas 621 (56.30%) and 294 (26.70%) received transplants from male and nulliparous female donors, respectively. HSCTs from parous female donors compared to male and nulliparous females were associated with a significantly higher incidence of grade III-IV acute (a) GVHD (55.27% vs. 11.34 and 10.84%) and extensive chronic (c) GVHD (64.32% vs. 15.52 and 13.65%), as well as lower relapse incidence (RI).

**Discussion:**

This study finds that while parous female donors are associated with higher incidences of grade III-IV aGVHD and extensive cGVHD post-allo-HSCT, the advantages, such as a lower RI, outweigh the risks. The results of our study provide valuable insights for donor selection.

## Introduction

Allogeneic hematopoietic stem cell transplantation (allo-HSCT) stands out as one of the most efficacious therapeutic modalities for individuals with hematological malignancies and bone marrow failure syndromes (1). Nevertheless, this treatment approach is associated with substantial morbidity and mortality (2). Donor choosing remains to play a pivotal role in transplantation due to its importance in post-HSCT outcomes. Major histocompatibility antigens (MHC) matching is crucial for appropriate donor selection. However, numerous criteria beyond HLA compatibility including, age, sex, ABO compatibility, and parity (i.e., the history and number of prior pregnancies), impact the selection of a suitable donor (3–5). Typically, donors who are HLA-identical siblings are favored. But, some patients may possess multiple siblings who are HLA-matched. In addition, unrelated donors (URD) are being extensively used for allo-HSCT and have shown similar long-term survival when compared to matched related donors (MRDs) (6–9). Therefore, it is crucial to comprehend the impact of donor-related factors beyond HLA matching on outcomes following SCT.

Donor parity is often a debated non-human leukocyte antigen (non-HLA) factor that affects the outcome of HSCT. Various research studies indicate that individuals receiving grafts from parous female donors exhibit a significantly greater incidence of acute or chronic graft versus host disease (aGVHD or cGVHD) when compared to recipients of male or nulliparous donors (2, 4, 5, 10–14). Pregnancy frequently results in alloimmunization of T and B cells through the exchange of cells between the mother and the fetus via the placenta. There is substantial evidence that maternal T cells that are alloimmune and specific to neonatal inherited paternal antigens (IPA) persist for a lengthy amount of time after delivery (15, 16). Furthermore, certain studies have described that male recipients might face even greater risk due to the female donor’s immune response to the H-Y antigen (2, 4, 13, 14). In contrast, some studies have not found any correlation between parity and the increased risk of developing GVHD (17).

In this investigation, we sought to determine the influence of donor parity on the incidence of high grade aGVHD and extensive cGVHD in a large homogeneously treated adult patients receiving an HLA-identical allo-HSCT.

## Materials and methods

### Ethical considerations and data collection

The current study was carried out in compliance with pertinent guidelines and regulations. Approval for this research was granted by the ethical committee of the Research Institute for Oncology, Hematology, and Cell Therapy (HORCSCT), as indicated by the reference number IR.TUMS.HORCSCT.REC.1400.023. All the participants submitted written informed consent, thereby authorizing the application of their data within the scope of the study. Patients’ and donors’ demographic, clinical, and laboratory data was gathered from their medical records using a checklist. The data was subsequently updated, and the patients were followed up until the end of 2022.

### Study design and inclusion criteria

This retrospective cohort study was conducted at Research Institute for Oncology, Hematology and Cell Therapy of Shariati Hospital, affiliated with Tehran University of Medical Sciences, Tehran, Iran. All adult patients presenting to our institution with acute myeloid leukemia (AML) and acute lymphoblastic leukemia (ALL) who underwent the first allo-HSCT in complete remission (CR) from an HLA-matched related donor following uniform busulfan (BU)-based myeloablative conditioning (MAC) regimen between Feb 2010 and Jan 2021 were included. Patients who received a graft of bone marrow or cord blood, those who underwent allo-HSCT from a matched unrelated donor, and those who received a reduced intensity conditioning regimen were excluded to make a more homogenous population and reduce confounding variables. The primary objective of this study was to investigate the predictive impact of donor parity on the incidence of grade III-IV aGVHD and extensive cGVHD, following HLA-identical allo-HSCT.

### Transplant procedure

Every recipient was given an identical MAC regimen, which involved administering either oral busulfan (Bu) at a dosage of 4 mg/kg/day or intravenous Bu (Busilvex) of 3.2 mg/kg/day between days -6 to -3, along with cyclophosphamide with a dose of 60 mg/kg/day on days -3 and -2. The prophylaxis for GVHD consisted of cyclosporine A (CyA) that was initiated intravenously at a dosage of 1.5 mg/kg/day on day -2, followed by 3 mg/kg/day from day +7 until oral tolerance was attained, and methotrexate (MTX) with a dose of 10 mg/m^2^ on day +1, followed by 6 mg/m^2^ on days +3, +6, and +11.

All the patients received acyclovir, fluconazole, and trimethoprim/sulfamethoxazole for prophylaxis against herpes simplex virus (HSV), candida, and Pneumocystis jirovecii infections. Cytomegalovirus (CMV) reactivation was monitored through biweekly screening using DNA polymerase chain reaction. Ganciclovir was given as the preemptive treatment when CMV was reactivated.

### Outcomes and definitions

The primary endpoints were grade III-IV aGVHD (at day-100) and 1-year extensive cGVHD. The secondary endpoints encompassed 5-year relapse incidence (RI), GVHD-free relapse-free survival (GRFS), and overall survival (OS) rates. GRFS was denoted as survival without grade III-IV aGVHD, extensive cGVHD, or relapse (18) and OS was characterized as the time until death. Diagnosis and grading of acute and chronic GVHD were under Glucksberg’s criteria (18) and the National Institutes of Health consensus guidelines (19).

### Statistical analysis

The between-group comparison of the demographic, clinical, and laboratory data was performed through the Mann-Whitney U and chi-squared tests for continuous and categorical variables, respectively. The median follow-up time was determined using the reverse Kaplan-Meier method. Also, the Kaplan-Meier method was implemented to estimate GRFS and OS, and their comparison was carried out among various categories of each covariate using the log-rank X² test. Moreover, the Fine and Gray tests were used to calculate and compare the cumulative incidences (CIs) of grade III-IV aGVHD, extensive cGVHD, and RI.

Using the Cox proportional hazard regression model, multivariable analyses were conducted to assess the effects of donor parity on outcomes considering confounding factors. The recipient and donor’s age, sex matching, primary disease, and pre-transplant remission status were covariates that included in univariable analyses. Only variables that demonstrated a p-value below 0.2 in the univariable analyses were incorporated into the multivariate analysis. A p-value of less than 0.05 was considered as the statistical significance of the entire analyses. All statistical analyses were performed using STATA version 17 (StataCorp, LP, College Station, TX, USA).

## Results

1103 patients made up this study, of whom 438 (39.70%) were female and 665 (60.30%) were male. 188 (17%) of these patients had transplants from female parous donors, whereas 621 (56.30%) and 294 (26.70%) of these patients received transplants from male and nulliparous female donors, respectively. The donors’ and recipients’ mean ages were 33.51 and 33.69 years, respectively, with a median age of 32 for both groups. Furthermore, as the primary disease, 415 (37.62%) of the recipients had ALL, and 688 (62.38%) had AML. All patients have been followed up for a median of 73.59 (95%CI: 69.78– 75.89) months. **Table 1** summarizes the patients’ and donors' baseline characteristics.

**Table 1:** Baseline characteristics of donors and recipients.

Characteristic		Donor Sex/Parity			
		Parous female	Male	Nulliparous female	Total
Donor age, n (%)	< 32	36 (6.90%)	283 (53.90%)	206 (39.20%)	525 (47.60%)
	≥ 32	152 (26.30%)	338 (58.50%)	88 (15.20%)	578 (52.40%)
Recipients’ age, n (%)	< 32	53 (10.20%)	283 (54.20%)	186 (35.60%)	522 (47.30%)
	≥ 32	135 (23.20%)	338 (58.20%)	108 (18.60%)	581 (52.70%)
Recipients’ sex, n (%)	Female	80 (18.30%)	238 (54.30%)	120 (27.40%)	438 (39.70%)
	Male	108 (16.20%)	383 (57.60%)	174 (26.20%)	665 (60.30%)
Primary disease, n (%)	ALL	51 (12.30%)	221 (53.30%)	143 (34.50%)	415 (37.62%)
	AML	137 (19.90%)	400 (58.10%)	151 (21.90%)	688 (62.38%)
ABO matching, n (%)	Matched	104 (15.71%)	387 (58.46%)	171 (25.83%)	662 (60%)
	Minor mismatch	42 (22.82%)	94 (51.09%)	48 (26.09%)	184 (16.7%)
	Major mismatch	35 (18.14%)	96 (49.74%)	62 (32.12%)	193 (17.5%)
	Bidirectional	7 (10.94%)	44 (68.75%)	13 (20.31%)	64 (5.8%)
Disease status, n (%)	CR1	148 (17.67%)	471 (56.20%)	219 (26.13%)	838 (76%)
	CR≥ 2	35 (13.83%)	145 (57.31%)	73 (28.86%)	253 (22.9%)
Graft cell dose, mean ± SD	CD34 cells	5.29 ± 2.53	6.04 ± 6.44	6.26 ± 20.99	5.97 ± 11.84
	CD3 cells	292.39 ± 83.27	278.62 ± 101.91	307.20 ± 122.48	288.54 ± 105.71
Total, n (%)		188 (17.00%)	621 (56.30%)	294 (26.70%)	1103 (100%)

AML indicates acute myeloid leukemia; ALL, acute lymphoblastic leukemia, CR, complete remission.

**Figure 1:**
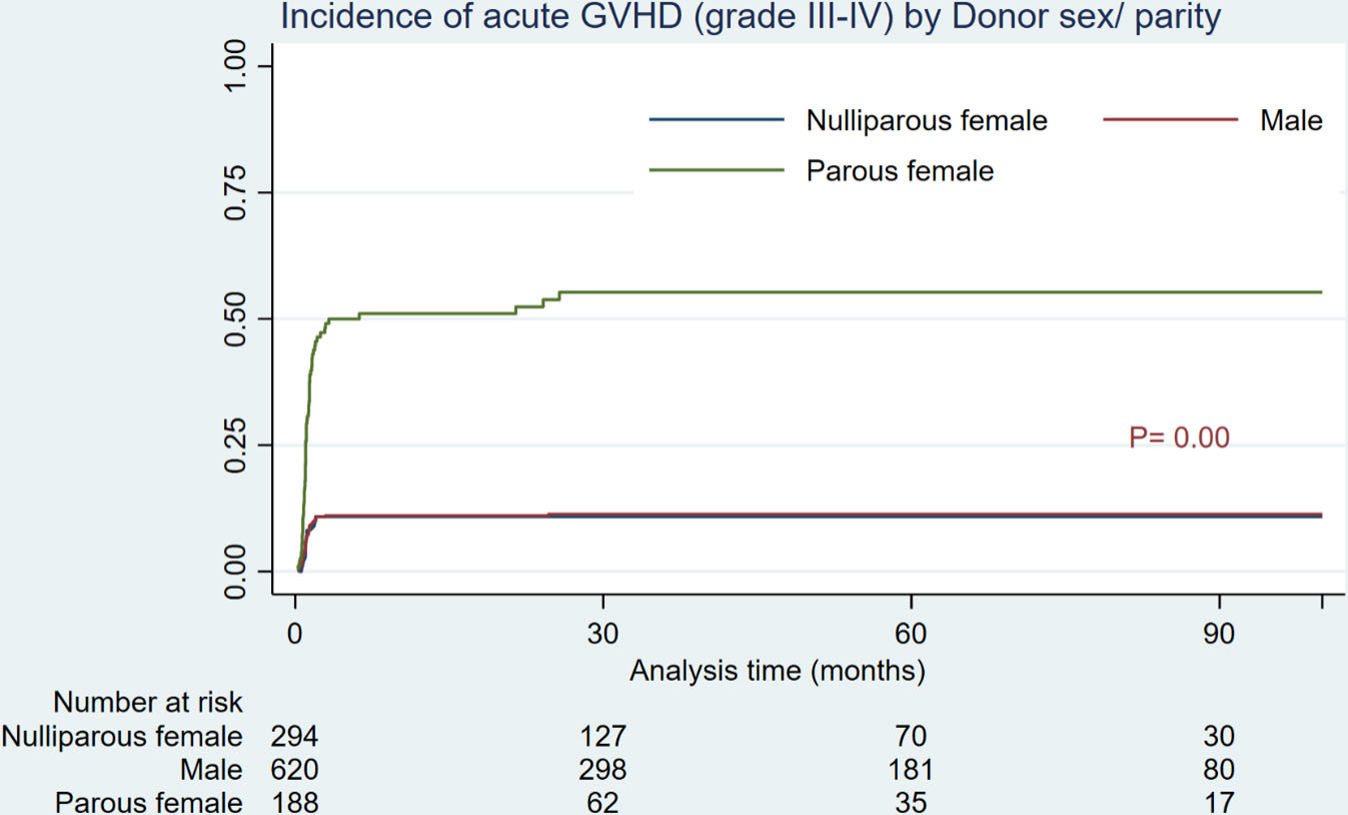
Cumulative incidence of grade III-IV acute GVHD by donor sex/parity.

**Figure 2:**
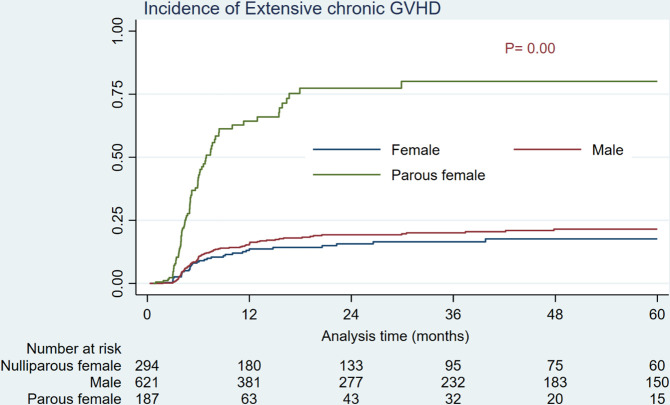
Cumulative incidence of extensive chronic GVHD by donor sex/parity.

As shown in **Table 2** and **Figure 1**, HSCTs from parous female donors were associated with a significantly higher incidence of grade III-IV aGVHD compared to male and nulliparous female donors (55.27% vs 11.34 and 10.84%, *P*= 0.00). Additionally, parous female donors showed a substantially higher incidence of extensive cGVHD (64.32% vs 15.52 and 13.65%, *P*= 0.00) than men and nulliparous female donors (**Table 2**, **Figure 2**).

**Table 2:** Post-transplant outcomes according to donor sex/parity.

	Donor sex/ parity	Probability (%)	95% CI	*P*
**Grade III-IV aGVHD**	Parous female	55.27	43.96-69.00	0.00
	Male	11.34	8.88-14.00	
	Nulliparous female	10.84	7.58-16.00	
**Extensive cGVHD**	Parous female	64.32	50.67-82.00	0.00
	Male	15.52	12.38-19.00	
	Nulliparous female	13.65	9.57-19.00	
**GRFS**	Parous female	11.48	7.19-16.87	0.000
	Male	41.41	37.32-45.43	
	Nulliparous female	36.01	30.17-41.87	
**RI**	Parous female	21.26	14.22-31.77	0.00
	Male	39.24	33.53-45.93	
	Nulliparous female	46.51	37.35-57.91	
**OS**	Parous female	49.17	41.57-56.31	0.039
	Male	56.32	52.12-60.29	
	Nulliparous female	48.61	42.48-54.45	

aGVHD indicates acute graft-versus-host disease; cGVHD, chronic graft-versus-host disease; CI, confidence interval; CR2, second complete remission; GFRS, graft-versus-host disease free relapse free survival; OS, overall survival; RI, relapse incidence.

In univariate analyses, factors apart from the parity status that were associated with an increased risk of grade III-IV aGVHD and extensive cGVHD were donor age (≥ 32 vs. < 32) and sex (male vs. female), recipient age (≥ 32 vs. < 32), and primary disease (AML vs. ALL). However, multivariate analysis showed that the greater age of the donor (HR= 1.53, P= 0.03), and parity history (HR= 3.90, P= 0.00) remained significant predictors of grade III-IV aGVHD; while, the parous female donors posed the sole significant risk for extensive cGVHD (HR= 4.62, P= 0.00) (**Table 3**).

**Table 3:** Multivariable Cox regression analyses for the outcomes.

Outcome	Variable		*P*	HR	95% CI
**Grade III-IV aGVHD**	Donor age (≥ 32 vs. < 32)		0.031	1.534	1.040-2.264
	Donor sex (male vs. female)		0.791	0.942	0.605-1.467
	Parous female donor vs. not		0.000	3.908	2.470-6.184
	Recipient age (≥ 32 vs. < 32)		0.907	0.979	0.684-1.401
	Primary disease (AML vs. ALL)		0.900	1.021	0.736-1.417
**Extensive cGVHD**	Donor age (≥ 32 vs. < 32)		0.939	1.013	0.724-1.417
	Donor sex (male vs. female)		0.499	1.146	0.772-1.701
	Parous female donor vs. not		0.000	4.623	3.024-7.067
	Recipient age (≥ 32 vs. < 32)		0.439	1.137	0.821-1.575
	Primary disease (AML vs. ALL)		0.202	1.219	0.899-1.651
**RI**	Parous female donor vs. not		0.020	0.616	0.410-0.928
	Recipient age (≥ 32 vs. < 32)		0.688	0.950	0.741-1.219
	Recipient sex (male vs. female)		0.011	1.388	1.079-1.785
	Primary disease (AML vs. ALL)		0.000	0.583	0.457-0.745
	Disease status (≥ CR2 vs. CR1)		0.000	1.861	1.449-2.391
**GRFS**	Donor age (≥ 32 vs. < 32)		0.004	1.305	1.089-1.565
	Donor sex (male vs. female)		0.207	0.887	0.737-1.068
	Parous female donor vs. not		0.000	2.266	1.802-2.850
	Recipient age (≥ 32 vs. < 32)		0.563	0.949	0.796-1.132
	Primary disease (AML vs. ALL)		0.023	0.834	0.713-0.975
**OS**	Donor age (≥ 32 vs. < 32)		0.001	1.405	1.141-1.729
	Parous female donor vs. not		0.147	1.189	0.941-1.502
	Recipient age (≥ 32 vs. < 32)		0.547	0.938	0.762-1.155
	Recipient sex (male vs. female)		0.080	1.179	0.981-1.418
	Primary disease (AML vs. ALL)		0.000	0.644	0.535-0.775
	Disease status (≥ CR2 vs. CR1)		0.000	1.592	1.309-1.937

aGVHD indicates acute graft-versus-host disease; AML, acute myeloid leukemia; ALL, acute lymphoblastic leukemia; cGVHD, chronic graft-versus-host disease; CI, confidence interval; CR2, second complete remission; GFRS, graft-versus-host disease free relapse free survival; HR, hazard ratio; OS, overall survival; RI, relapse incidence.

**Figure 3:**
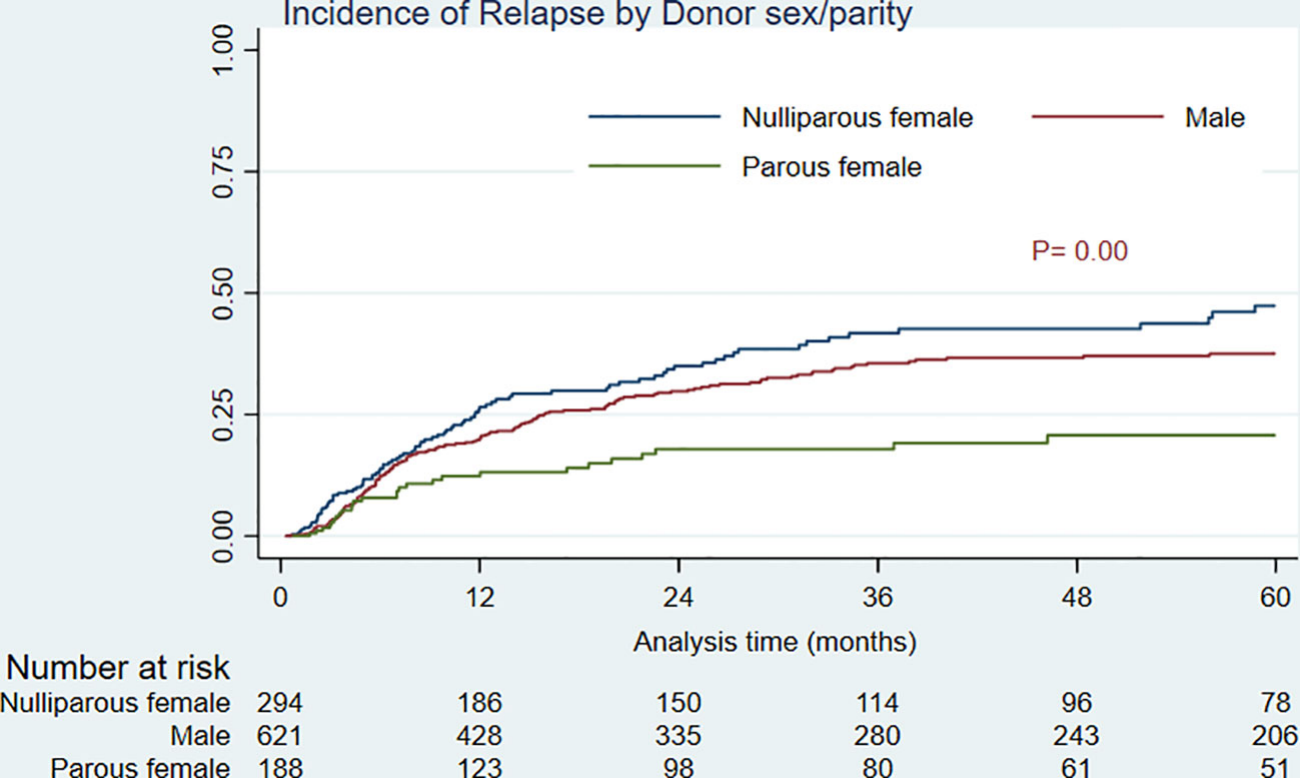
Relapse incidence by donor sex/parity.

As shown in **Table 2** and **Figure 3**, the 5-year RI for parous female transplant recipients was significantly lower than for male and nulliparous female recipients (21.26% versus 39.24% and 46.51%, *P*= 0.00). Additional factors associated with higher RI in univariate analysis were recipient age and sex (male vs. female), as well as primary disease of AML and disease status of second complete remission and above (≥ CR2) before transplant. In multivariate analysis (**Table 3**), male recipients and disease status of ≥ CR2 were the predictive hazard factors (HR= 1.38, P= 0.01 and HR= 1.86, P= 0.00, respectively), whereas parous donors and primary disease of AML were the protective factors against RI (HR= 0.61, P= 0.02 and HR= 0.58, P= 0.00, respectively). Furthermore, RIs for patients transplanted from all three types of donors at CR ≥ 2 were significantly escalated compared to recipients at CR1 (results not shown).

HSCTs from females of parous type were also associated with significantly poorer GRFS of 5 years compared to male and nulliparous females (11.48% vs 41.41% and 36.01%, *P*= 0.00). Other characteristics associated with GRFS in univariate analysis were donor age and sex, recipient age, and primary disease. In Multivariate analysis, AML as the primary disease showed a significantly better probability of 5-year GRFS compared to ALL (HR= 0.83, P= 0.02), while the donor age of ≥ 32 and parous female donor significantly reduced the GRFS at 5-year (HR= 1.30, P= 0.00 and HR= 2.26, P= 0.00, respectively).

On the other hand, the 5-year OS among individuals transplanted from parous female donors was comparable to those from nulliparous females but significantly reduced than recipients of male donors (**Table 2**). Donor age, recipient age and sex, disease status, and primary disease were all significantly associated with OS in univariate analysis. Moreover, in multivariate analysis, primary disease of AML (HR= 0.64, P= 0.00) was shown to be the only predictive factor for better OS, whereas donor’s age of ≥ 32 and disease status of ≥ CR2 were significant predictors for a lowered OS (HR= 1.40 and HR= 1.59, respectively) (**Table 3**).

Considering sex matches, female donors for male recipients (F-M) were found to be associated with significantly higher incidences of grade III-IV aGVHD and extensive cGVHD when compared to the other sex matches that were combined into one group. However, there were no significant differences in 5-year RI (results not shown).

## Discussion

Suitable donor selection is vital for reducing risks and improving outcomes, constituting an essential part of the clinical transplantation procedure. Numerous research has been conducted to evaluate the predictors of outcomes following allo-HSCT. Among the variables analyzed, donor parity is an aspect that has got the least attention, and its impact on HSCT outcomes and GVHD is disputed. Our study aimed to investigate the outcomes of allo-HSCT over a decade-long period, with a specific focus on grafts obtained from female donors who had a history of previous pregnancy.

We observed that grade III-IV aGVHD and extensive cGVHD incidences were significantly higher in recipients who received the graft from parous female donors compared to the grafts from male and nulliparous donors. This finding lends credence to the concept that an alloimmunization induced during pregnancy may result in prolonged immune activation, which in turn might elevate the risk of acute and chronic GVHD. In contrast, the 5-year RI in recipients of parous female donors was approximately half that of recipients of male or nulliparous donors. Donor parity was also found to have no significant effect on survival, suggesting that the predictive effect of donor parity on higher incidences of grade III-IV aGVHD and extensive cGVHD can be compensated by the advantage of increasing graft-versus-tumor effect and a lower risk of relapse, leading to no noticeable impact on survival. However, the 5-year GRFS for allo-HSCT from parous donors was much lower compared to other donor types.

Reports regarding the impacts of donor parity on HSCT outcomes need to be more consistent. The study of Flowers et al. (20) on the patients with aplastic anemia who received the HSCT from HLA-identical siblings described the donor parity as a significant risk factor for the incidence of grade II-IV aGVHD compared to nulliparous female donors (RR= 2.5, P= 0.02). The research conducted by Loren et al. (2) also displayed an elevated risk for aGVHD in HSCT from parous women (unadjusted HR= 1.16, P= 0.04) compared to male or nulliparous female donors. In our study, the robust predictive impact of donor parity on grade III-IV aGVHD incidence, along with donor age, persisted regardless of other possible confounding factors (HR= 3.90, P= 0.00). On the other hand, Przepiorka et al. (17) observed that the gestation history of the donor did not affect the hazard for grade II-IV aGVHD incidence in recipients of HLA-matched related donors. However, donor parity along with donor-recipient sex mismatch appeared to be significant risk factors for aGVHD of grades II-IV (P= 0.001) in the study by Nash et al. (21). Another study conducted by Gale et al. (11) also showed that compared with other donor-recipient sex combinations, the female-to-male combination was associated with significantly higher incidence of moderate to severe aGVHD especially in case of parous female donors.

Similar to the results of our analysis, the donor parity was not a significant risk factor for poor survival in the multivariate Cox regression model conducted by Flowers et al. (RR= 1.6, P= 0.30); however, they showed the survival rate was worse among the patients transplanted from parous females than the recipients from nulliparous females (47% vs 68%) (20). The analysis by Loren et al. (2) also did not find any effect of donor parity on OS among the patients who underwent the HSCT from HLA-identical siblings in the multivariate model fitted.

Data provided by the Center for International Blood and Marrow Transplant Research (CIBMTR) in a large cohort study revealed that donor sex, donor pregnancy history, and recipient age were significantly associated with the onset of cGVHD (2), while donor parity was the sole variable that significantly influenced the risk of extensive cGVHD in our study. Regarding the augmented risk of cGVHD, the predictive effects of other factors such as aGVHD grades I-IV, males receiving grafts from allo-immunized females, and donors age have all been recognized significant in the multivariate analyses by previous studies (10, 22–24).

Regarding the relapse incidence, we found donor parity was significantly associated with decreased relapse risk. This was inconsistent with the results obtained by Loren et al. (2), who failed to identify any relationship between donor parity and RI. In the case of GFRS, we did not find any study in the literature to assess the effect of donor parity post-HSCT.

The discrepancy between our findings and the results of other studies can be due to the different protocols and wide heterogenicity such as sources of graft, conditioning and GVHD prophylaxis regimens, as well as demographic characteristics of the donors and recipients. This study has several benefits and limitations. As an advantage, we selected a homogenous population of patients to minimize the effects of potential confounding variables as few as possible. For this goal, all included patients had undergone the HSCT from the peripheral blood as the single source of the graft, together with identical Bu-based MAC and GVHD prophylaxis regimens. However, the study was limited by its retrospective design, dearth of data regarding immune reconstitution, and absence of information regarding cytogenetic or molecular examinations. A further limitation is that the study’s data was restricted to the donor’s parity evaluation and lacked information about the number and sex of the children.

## Conclusion

This study casts light on the influence of donor parity on outcomes following HLA-identical allogeneic HSCT. However, our data showed that the predictive impact of donor parity on higher incidences of grade III-IV aGVHD and extensive cGVHD can be counterbalanced by the benefit of increasing graft-versus-tumor effect and a lower risk of relapse, resulting in no significant effect on survival, although it led to poorer GRFS. The findings highlight the importance of non-HLA factors in shaping GVHD incidence, relapse rates, and overall survival. This information equips clinicians with valuable insights for making informed decisions about donor choice and effectively managing potential GVHD risks.

## Data availability statement

The raw data supporting the conclusions of this article will be made available by the authors, without undue reservation. The corresponding author can provide the datasets created during the current investigation upon reasonable request.

## Ethics statement

The studies involving humans were approved by Research Institute for Oncology, Hematology, and Cell Therapy (HORCSCT), Tehran University of Medical Sciences, Tehran, Iran. The studies were conducted in accordance with the local legislation and institutional requirements. The participants provided their written informed consent to participate in this study.

## Author contributions

MA: Writing – original draft, Data curation. MB: Conceptualization, Formal analysis, Methodology, Supervision, Writing – review & editing. TB: Conceptualization, Supervision, Investigation, Writing – review & editing. SR: Data curation, Writing – review & editing. HKF: Data curation, Writing – review & editing. SAM: Data curation, Writing – review & editing. STS: Data curation, Writing – review & editing. MA: Data curation, Writing – review & editing. PS: Writing – review & editing. MV: Data curation, Project administration, Writing – review & editing.
