# Corrigendum: Determining the predictive impact of donor parity on the outcomes of human leukocyte antigen matched hematopoietic stem cell transplants: a retrospective, single-center study

**DOI:** 10.3389/fonc.2024.1397607

**Published:** 2024-04-18

**Authors:** Mojtaba Azari, Maryam Barkhordar, Tanaz Bahri, Soroush Rad, Hosein Kamranzadeh Fumani, Seied Asadollah Mousavi, Sahar Tavakoli Shiraji, Morteza Azari, Parisa Shafaroudi, Mohammad Vaezi

**Affiliations:** ^1^ Cell Therapy and Hematopoietic Stem Cell Transplantation Research Center, Tehran University of Medical Sciences, Tehran, Iran; ^2^ Research Institute for Oncology, Hematology, and Cell Therapy, Tehran University of Medical Sciences, Tehran, Iran; ^3^ Hematology, Oncology and Stem Cell Transplantation Research Center, Tehran University of Medical Sciences, Tehran, Iran; ^4^ Hematologic Malignancies Research Center, Tehran University of Medical Science, Tehran, Iran

**Keywords:** graft-versus-host disease (GVHD), hematopoietic stem cell transplantation (HSCT), donor parity, overall survival, relapse incidence

In the published article, there was an error in **Table 1** as published. In **Table 1**, Characteristic column, Primary disease classification section (4th row, 2nd column), “AML” and “ALL” have been miswritten interchangeably. The corrected **Table 1** and its caption appear below.

**Table 1:** Baseline characteristics of donors and recipients.

Characteristic		Donor Sex/Parity			
		Parous female	Male	Nulliparous female	Total
**Donor age, n (%)**	**< 32**	**36 (6.90%)**	**283 (53.90%)**	**206 (39.20%)**	**525 (47.60%)**
	**≥ 32**	**152 (26.30%)**	**338 (58.50%)**	**88 (15.20%)**	**578 (52.40%)**
**Recipients’ age, n (%)**	**< 32**	**53 (10.20%)**	**283 (54.20%)**	**186 (35.60%)**	**522 (47.30%)**
	**≥ 32**	**135 (23.20%)**	**338 (58.20%)**	**108 (18.60%)**	**581 (52.70%)**
**Recipients’ sex, n (%)**	**Female**	**80 (18.30%)**	**238 (54.30%)**	**120 (27.40%)**	**438 (39.70%)**
	**Male**	**108 (16.20%)**	**383 (57.60%)**	**174 (26.20%)**	**665 (60.30%)**
**Primary disease, n (%)**	**ALL**	**51 (12.30%)**	**221 (53.30%)**	**143 (34.50%)**	**415 (37.62%)**
	**AML**	**137 (19.90%)**	**400 (58.10%)**	**151 (21.90%)**	**688 (62.38%)**
**ABO matching, n (%)**	**Matched**	**104 (15.71%)**	**387 (58.46%)**	**171 (25.83%)**	**662 (60%)**
	**Minor mismatch**	**42 (22.82%)**	**94 (51.09%)**	**48 (26.09%)**	**184 (16.7%)**
	**Major mismatch**	**35 (18.14%)**	**96 (49.74%)**	**62 (32.12%)**	**193 (17.5%)**
	**Bidirectional**	**7 (10.94%)**	**44 (68.75%)**	**13 (20.31%)**	**64 (5.8%)**
**Disease status, n (%)**	**CR1**	**148 (17.67%)**	**471 (56.20%)**	**219 (26.13%)**	**838 (76%)**
	**CR≥ 2**	**35 (13.83%)**	**145 (57.31%)**	**73 (28.86%)**	**253 (22.9%)**
**Graft cell dose, mean ± SD**	**CD34 cells**	**5.29 ± 2.53**	**6.04 ± 6.44**	**6.26 ± 20.99**	**5.97 ± 11.84**
	**CD3 cells**	**292.39 ± 83.27**	**278.62 ± 101.91**	**307.20 ± 122.48**	**288.54 ± 105.71**
**Total, n (%)**		**188 (17.00%)**	**621 (56.30%)**	**294 (26.70%)**	**1103 (100%)**

AML indicates acute myeloid leukemia; ALL, acute lymphoblastic leukemia, CR, complete remission.

In the published article, there were some errors in the Text of Abstract, **Methods and Materials**, and **Results** section. In the final manuscript we updated the Tables and Figures according to our final analysis. However, in the text of the manuscript in some places (mainly Multivariable Cox regression analyses for the outcomes in the Results section) we did not update the text according to our final Analysis, Tables, and Figures.

A correction has been made to Abstract section, **Results** subsection. This sentence previously stated:

“188 (17.04%) had…”

The corrected sentence appears below:

“188 (17%) had…”

A correction has been made to **Methods and Materials** section, **Outcomes and definitions** subsection, first paragraph.

This sentence previously stated: “3-year extensive cGVHD”

The corrected sentence appears below:

“1-year extensive cGVHD”

A correction has been made to **Results** section, first paragraph, line 2. This sentence previously stated: “188 (17.04%) of these patients…”

The corrected sentence appears below:

“188 (17%) of these patients…”

A correction has been made to **Results** section, third paragraph. This sentence previously stated: “(HR= 1.48, P= 0.02)”

The corrected sentence appears below:

“(HR= 1.53, P= 0.03)”

A correction has been made to **Results** section, third paragraph. This sentence previously stated: “(HR= 4.14, P= 0.00)”

The corrected sentence appears below:

“(HR= 3.90, P= 0.00)”

A correction has been made to **Results** section, third paragraph. This sentence previously stated: “(HR= 4.46, P= 0.00)”

The corrected sentence appears below:

“(HR= 4.62, P= 0.00)”

A correction has been made to **Results** section, fourth paragraph. This sentence previously stated: “Additional factors associated with higher RI in univariate analysis were recipient age and sex (male vs. female), as well as disease status of second complete remission and above (≥ CR2) before transplant.”

The corrected sentence appears below:

“Additional factors associated with higher RI in univariate analysis were recipient age and sex (male vs. female), as well as primary disease of AML and disease status of second complete remission and above (≥ CR2) before transplant.”

A correction has been made to **Results** section, fourth paragraph. This sentence previously stated: “(HR= 1.38, P= 0.01 and HR= 1.87, P= 0.00, respectively)”

The corrected sentence appears below:

“(HR= 1.38, P= 0.01 and HR= 1.86, P= 0.00, respectively)”

A correction has been made to **Results** section, fourth paragraph. This sentence previously stated: “(HR= 0.57, P= 0.00 and HR= 0.61, P= 0.02, respectively)”

The corrected sentence appears below:

“(HR= 0.61, P= 0.02 and HR= 0.58, P= 0.00, respectively)”

A correction has been made to **Results** section, fifth paragraph. This sentence previously stated: “(HR= 0.81, P= 0.01)”

The corrected sentence appears below:

“(HR= 0.83, P= 0.02)”

A correction has been made to **Results** section, fifth paragraph. This sentence previously stated: “(HR= 1.25, P= 0.01 and HR= 2.49, P= 0.00, respectively)”

The corrected sentence appears below:

“(HR= 1.30, P= 0.00 and HR= 2.26, P= 0.00, respectively)”

A correction has been made to **Results** section, sixth paragraph. This sentence previously stated: “(HR= 0.63, P= 0.00)”

The corrected sentence appears below:

“(HR= 0.64, P= 0.00)”

A correction has been made to **Results** section, sixth paragraph. This sentence previously stated: “(HR= 1.44 and HR= 1.61, respectively)”

The corrected sentence appears below:

“(HR= 1.40 and HR= 1.59, respectively)”

As published, there were errors in the **Discussion** section. As the results obtained by Kumar et al. may be misinterpreted by us, we have deleted reference 21 and its results from the mentioned parts in our **Discussion** section of published article. A correction has been made to the **Discussion** section, third paragraph. This sentence previously stated:

“Przepiorka et al. (17) and Kumar et al. (21) observed that the gestation history of the donor did not affect the hazard for grade II-IV aGVHD incidence in recipients of HLA-matched related donors.”

The corrected sentence appears below:

“Przepiorka et al. (17) observed that the gestation history of the donor did not affect the hazard for grade II-IV aGVHD incidence in recipients of HLA-matched related donors.”

A correction has been made to the **Discussion** section, paragraph 4. The sentence previously stated: “The analysis by Loren et al. (2) and Kumar et al. (21) also did not find any effect of donor parity on OS among the patients who underwent the HSCT from HLA-identical siblings in the multivariate model fitted.”

The corrected sentence appears below:

“The analysis by Loren et al. (2) also did not find any effect of donor parity on OS among the patients who underwent the HSCT from HLA-identical siblings in the multivariate model fitted.”

These errors do not change the scientific conclusions of the article in any way.

The authors apologize for these errors and state that this does not change the scientific conclusions of the article in any way. The original article has been updated.

